# Hippocampal cellular loss after brief hypotension

**DOI:** 10.1186/2193-1801-2-23

**Published:** 2013-01-25

**Authors:** Rafael E Chaparro, Carolina Quiroga, Gerardo Bosco, Diana Erasso, Alessandro Rubini, Devanand Mangar, Andrea Parmagnani, Enrico M Camporesi

**Affiliations:** Department of Molecular Pharmacology and Physiology, University of South Florida, 12901 Bruce B Downs Blvd, Tampa, FL 33612 USA; Department of Neurosciences, University of South Florida, 12901 Bruce B Downs Blvd, Tampa, FL 33612 USA; Tampa General Hospital, 1, Tampa General Hospital Circle, Tampa, FL 33606 USA; Department of Biomedical Sciences, Physiology Lab, University of Padova, Via Marzolo 3, Padova, 35131 Italy

**Keywords:** Hypotension, Rats, Hemorrhagic shock model, Neuronal damage

## Abstract

Brief episodes of hypotension have been shown to cause acute brain damage in animal models. We used a rat hemorrhagic shock model to assess functional outcome and to measure the relative neuronal damage at 1, 4 and 14 days post-injury (3 min of hypotension). All rats underwent a neurological assessment including motor abilities, sensory system evaluation and retrograde memory at post-hypotensive insult. Brains were harvested and stained for Fluorojade C and Nissl. Stereology was used to analyze Fluorojade C and Nissl stained brain sections to quantitatively detect neuronal damage after the hypotensive insult. Statistical analysis was performed using Graphpad Prism 5 with the Bonferroni test at a 95% confidence interval after ANOVA. A Mixed Effect Model was used for the passive avoidance evaluation. Stereologically counted fluorojade positive cells in the hippocampus revealed significant differences in neuronal cell injury between control rats and rats that received 3 min of hypotension one day after insult. Quantification of Nissl positive neuronal cells showed a significant decrease in the number hippocampal cells at day 14. No changes in frontal cortical cells were evident at any time, no significative changes in neurological assessments as well. Our observations show that brief periods of hemorrhage-induced hypotension actually result in neuronal cell damage in Sprague–Dawley rats even if the extent of neuronal damage that was incurred was not significant enough to cause changes in motor or sensory behavior.

## Background

Maintenance of adequate blood flow to the brain is necessary in the course of general anesthesia in order to assure safe recovery and normal brain function after surgical intervention (Rubio et al. 2008; Moritz et al. 2007). Predictable models of neuronal loss after progressive low blood pressure insults have been developed (Yamauchi et al. 1990,1991) For instance, Yamauchi and colleagues described selective progressive damage to regions of the brain after two or three minute episodes of profound hypotension (low blood pressure, 25 mmHg) one week after the hypotensive insult (Yamauchi et al. 1990,1991). These changes were attributed to neuronal necrosis (Fukuda and Warner 2007). However, this study did not measure functional behavior after recovery from hypotension. Cognitive dysfunction has been linked with hypotension (Duschek et al. 2007 Wharton et al. 2006), especially in elderly patients (Zuccala et al. 2001; Qiu et al. 2005). Several studies have studied the relationship between hypotension during surgery and neurological performance after surgery but so far a clear link has not been found (Williams-Russo et al. 1999). The largest study that evaluated this matter was “The International Study of Postoperative Cognitive Dysfunction” but no association between surgical blood pressure and postoperative cognitive function was found (Moller et al. 1998). In contrast, other researchers have found a link between postoperative cognitive function and blood pressure during surgery (Schutz et al. 2006; Yocum et al. 2009).

With this in mind we designed an animal study to test the hypothesis that hypotensive episodes during surgery may cause long-term functional alterations. A secondary objective of this study was to characterize an animal model of hypotension that would produce consistent cerebral damage that could be used for future analyses.

## Materials and methods

### Regulations

This study was approved by the division of Comparative Medicine at the University of South Florida (USF). All experiments were done following IACUC guidelines. Male Sprague Dawley rats were separated into four groups as follows; groups 1, 2 and 3 received 3 min of hypotension - 1 min every hour - and were evaluated at 1, 4 and 14 days, respectively. An additional group of rats, group 4, received a sham operation (Table 1).

**Table 1:** **Physiological variables**

	Group 1	Group 2	Group 3
	Euthanized at day 1	Euthanized at day 4	Euthanized at day 14
	(n = 7)	(n = 6)	(n = 5)
Initial weight (g)	352 ± 11	334 ± 12	344 ± 22
Final weight (g)	349 ± 13	362 ± 29	380 ± 39
Initial temp (°F)	94.8 ± 2.2	93.02 ± 2.6	92.58 ± 4.3
Final Temp (°F)	89.6 ± 7.5	91.88 ± 2.6	88.8 ± 3.1
First MAP (mmHg)	19 ± 2	20 ± 2	19 ± 2
Second MAP (mmHg)	19 ± 1	19 ± 1	20 ± 1
Third MAP (mmHg)	20 ± 2	18 ± 1	19 ± 1
Initial HR (bpm)	254 ± 49	271 ± 50	248 ± 61
Final HR (bpm)	267 ± 49	233 ± 59	250 ± 54
Initail Hb. Saturation	97 ± 2	94 ± 6	95 ± 4
Final Hb. Saturation	95 ± 5	94 ± 4	98 ± 7

Values are mean ± SE. MAP = Mean Arterial Pressure, HR = Heart rate, #To = Temperature, BPM = Beats Per Minute, Hb = Hemoglobin, F° = Fahrenheit. No significant differences were found.

### Rats

Male Sprague Dawley rats aged between 60 and 90 days with weights between 250 and 350g from Harlan Laboratories (Indianapolis, IN) were used. Upon arrival to the USF College of Medicine Vivarium, rats were housed in a climate controlled room in plastic cages in groups of two with free access to water and food and were left in quarantine for one week before the experiment took place.

### Neurological assessment

The Neurological state of the rat was assessed using the forty-eight point scale (Yokoo et al. 2004). The test was done at 2 different time points. The first evaluation was the day before surgery to assure that all rats were neurologically intact. Any rat that demonstrated a neurological deficit was not used for the study. No rats were withdrawn from the study under those criteria. The second evaluation was completed before tissue collection. The passive avoidance test was used in order to evaluate memory function following hypotension as described by Saporta (1999). Habituation is the first step. The animal was placed on a platform in a plexiglass box. The amount of time that the rat remained on the platform before they stepped down was measured. The following day, the rats were placed on the platform and received an electric shock (0.5 mA) for three seconds if they stepped down from the platform. After habituation and training, the rats learned to remain on the platform to avoid being shocked. All rats were trained before the hypotensive insult and were tested on the day before and the day of euthanasia. For all instances, the animal was placed on the platform and time to step-down was measured for a maximum of 5 min (300 seconds). Latencies greater than 300 seconds were assigned a default value of 300 seconds.

### Anesthesia

After the weight was recorded, the animal was anesthetized in an induction chamber with 5% Isoflurane in 100% Oxygen. After the rat was fully anesthetized we changed the animal from the induction chamber to a mask with 1 to 2% Isoflurane in 100% oxygen. The rats were continuously anesthetized during the three episodes of hypotension.

### Surgery

After shaving the neck, the area was cleaned with iodine and a medial linear incision was made. Plastic catheters were inserted in the jugular vein and both carotid arteries. The arterial lines were used for blood pressure quantification and blood aspiration. The venous line was used for blood reinfusion. The blood was rapidly aspirated until the mean arterial pressure (MAP) reached a point at or below 20 mmHg. The blood was withdrawn and reinfuse is between 10 to 20 seconds and the amount range was between 8 to 15 cc. The goal was not to reduce the circulating blood in an specific amount but achieve a blood pressure close to 20 mmHG. If the animal did not reach a MAP below 20 at any of the 3 episodes; it was excluded from the experiment. When MAP was below 20, the chronometer was set for 60 seconds. Between 8 cc to 15 cc of blood were taken to reach MAP of 20 or less. After the minute, the blood was reinfused using the venous catheter. During the procedure the rats were placed on a heated pad to prevent hypothermia. At the end of the third hypotensive episode the catheters were removed and skin was closed with a skin stapler. The rats recovered in a clean cage.

### Physiological parameters

The following variables were measured: Weight, temperature (measured with a rectal thermometer), hemoglobin saturation (Hb Sat), measured with a pulse oximeter, heart rate (HR), and blood pressure (BP) (obtained directly from an arterial catheter placed in the carotid artery) (SurgiVet Advisor Monitor, model number 92V303100 was used for Hb Sat, HR and BP). Readings were made before and after each ischemic event. Weight was measured right before the surgery and at euthanasia.

### Brain extraction and sectioning

The rats were euthanatized with an overdose of CO_2_ and the arterial tree was perfused with normal saline solution 0.9% followed by 4% paraformaldehyde. The brains were harvested, stored in plastic tubes with 4% paraformaldehyde for 24 hours, and then changed to 10%, 20% and 30% sucrose every 24 hours. The brains were sectioned at 40 μm with cryostat HM 550 from MICROM International, at a chamber temperature set at −22°C. A series of 5 coronal sections spaced approximately 960 microns apart were mounted for histopathology analysis and stained with Fluorojade C and Nissl. The brains were sectioned at 40 μm, the consecutive sections were collected in a 24 well plate previously fill out with PBS + azyde. The sections collected from wells # 1 and 13 were stain and mount on glass slides for counting. We used stereology (optical dissector) to count cells in frontal cortex and CA1 area of hypocampus.

### Fluoro jade C

Fluoro Jade C stain is commonly used in ischemia research to label degenerating neurons regardless of the insult. Fluoro Jade C analysis was used to quantify the number of degenerating cells in the cortex and CA1 area of the hippocampus; the method used has been described in detail (Ajmo et al. 2008). The sections were mounted on slides and air-dryed overnight; then, they were dipped in absolute ethyl alcohol 3 times, and 1 min in 70% ethyl alcohol and washed in running tap water 1 min, then stained with potassium permanganate to oxidize tissue for 15 min while shaking gently. 120 mg of potassium permanganate (KMnO_4_) 0.06% was diluted in 200 mL of PBS. At this point the slides were protected from light. Staining with Fluoro Jade C 0.001% for 30 min was followed by 3 changes of water for 1 min and air-dry overnight. The next day under the fume hood, they were cleared in xylene for 2 min, 3 times. Coverslip with DPX directly from xylene. Fluoro-Jade stock was made with 0.01% Fluoro Jade C in water (dilute 2 mg/ 20 ml). The working solution was made with 0.001% Fluoro-Jade C in 0.1% acetic acid, after diluting 20 ml stock with 180 mL of water plus 200 uL of acetic acid.

### Nissl

Nissl is a classic stain used for detection of Nissl bodies in the cytoplasm of cells, which will be stained purple-blue. Sections were mounted and air-dried on a slide warmer overnight. The following day the slides were dehydrated through 100% and 95% alcohol to distilled water. The slides were stained in 0.1% cresyl violet solution for 5 min and then rinsed with distilled water. The slides were then immersed in 95% ethyl alcohol for 30 min and posterior dehydrated in 100% alcohol 2 times for 5 min. Under the fume hood they were cleared in xylene for 2 min, 3 times and then immediately coverslipped with DPX.

### Statistical analysis

The data are presented as Mean ± SE. Counting of Fluoro Jade C positive cells and Nissl cells was completed using unbiased stereology (Optical fractionator) as described in by Mouton (2002). For this purpose a Stereologer from Stereology Resource Center, Chester, Maryland was used. The results are an estimate of the total number of cells. Neurological score data were also evaluated. One-way Analysis of Variance was followed by Bonferroni Multiple Comparison test. The results were analyzed using GraphPad Prism 5.0 for Mac. Passive avoidance was evaluated using a Mixed Effect Model. A p-value of <0.05 was considered statistically significant.

## Results and discussion

We designed the experiment with 40 rats, 7 of which died during surgery. Dead rats were not replaced (See Table 1). Physiological variables were considered and recorded. The baseline mean arterial pressure combining all the groups was 100.3 ± 42 mmHg, 94.3 ± 38 mmHg before the second insult and 102 ± 40 mmHg before the last insult. After the insult and blood re-infusion the mean arterial pressure was 87.7 ± 56.2 mmHg, 94.5 ± 57.2 mmHg and 92.7 ± 43.2 mmHg respectively. During the hypotension period, the blood was withdrawn until the MAP reach 20 or less. No statistically significant differences were found between the pre-operative values and the post-operative values. We also compared the weight before the ischemic event and at day 14 and did not find any statistically significant change (Table 1). In pilot studies we have tried 1 or 2 separate minutes of deep hypotension (MAP of 20 or less). The first insult that was successful in finding cellular lost was with three separate minutes of deep hypotension. We believe that accumulation of calcium is a key factor in developing the injury. Future experiments should focus on counting calcium levels after different number of 1 min insults to corroborate this hypothesis.

Neurological performance was evaluated in all rats. Behavior measurements on postoperative day 1, 4 and 14 were compared with control; at day one there were positive findings (unilateral palpebral ptosis). These findings were not present at day 4 or 14. Although there was some neurological impairment 24 hours after the injury, these changes were not statistically significant. Some rats showed some degree of paralysis immediately after the surgery but recovered by the time of the neurological evaluation. 10% of the animals showed temporary paralysis of the lower extremities, affected animals recovered in the next 24 hours after surgery. We believe that spinal cord ischemia could be the etiology of this finding. One of the animal models of spinal cord injury is achieved by temporal clipping of the thoracic aorta. We believe that the temporal paralysis that we have seen in some of this animals could be related to spinal cord ischemia.

**Figure 1:**
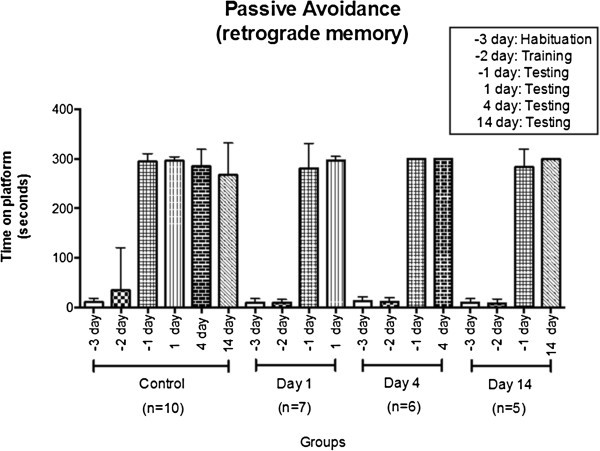
**All the animals only received one electric shock at day 2 before the injury.** There is no significant differences between control animals and ischemic animals at day 1, 4 or 14. Number of animals: total = 40; control = 10 animals; animals euthanized at day 1 = 7 alive (3 died); animals euthanized at day 4 = 6 alive (4 died); animals euthanized at day 14 = 5 alive (5 died). Total number of dead animals = 12.

Passive avoidance as described by Saporta (1999), was used to test the ability to recall old memories. During the habituation and training, rats learned to stay on the platform to avoid an electric shock (Figure 1).

We did not find any memory impairment in the rats after the surgery in comparison with the control rats.

**Figure 2:**
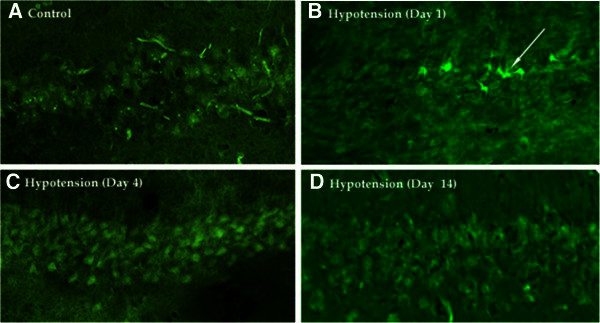
**Fluoro Jade C positive cells in Hypocampal CA1 area in rats subjected to 3 non consecutive minutes of hypotension.** Fluoro-Jade C positive cells in Hypocampal CA1 area in rats subjected to 3 non-consecutive minutes of hypotension. Fluoro-Jade C positive cells were absent in control (**A**), present at Day 1 after hypotension (**B**, arrow), and again absent at Day 4 (**C**) and Day 14 (**D**) after hypotension.

**Figure 3:**
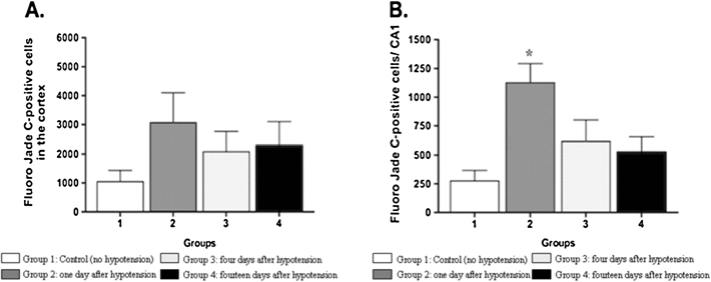
**FluroJade C positive labeled in the (A) cortex and (B) CA1 region, in control group, 1, 4 and 14 days following hypotension.** FluroJade C positive labeled was statistically significantly higher in the CA1 region of rats one day after hypotension as compared to control rats *p < 0.05. The fire shows the results from staining the brains with Fluoro Jade C. Panea **A** shows cell counting in frontal cortex. There is no significant difference in the number of Fluoro Jade C positive cells. This suggest that the cortex is not affected by the insult. Panel **B** shows cell counting in the CA1 area of the hyppocampus. The number of fluoro jade C positive cells increase significantly *(p < 0.05) one day after the injury in comparison with normal animals. This increase is not present at day 4 or 14th after the insult. This increase represents cell damage, by day 4 or 14th the cells have decided their fate and have survive of died. In either case they are not fluorescent anymore.

Stereology was used to estimate the total number of positive Fluoro Jade C cells in the frontal cortex and CA1 area of the hippocampus. No significant differences in cellular injury represented by numerous fluoro jade positive cortical cells were seen between control rats and rats that received 3 min of hypotension (p > 0.05). The pictures from Figure 2 shown the groups represented in Figure 3B. The first one is from an animal that received the sham operation, it shows a low number of fluoro jade positive cells, in contrast, 24 hours after the insult there is an increase in the number of cells that are positive for fluoro jade c (positive cells are green and bright like the one shown with the arrow). This means that since the cells are damage the stain was able to get into the genetic material. These changes are not noticeable at day 4 or 14. This can be explained because at this late time points the cells resolved the damage so they are either dead or they recovered from the injury. For this reason it was important to check for the number of live cells at this time points. We found congruent results. At day 1 and 4th the number of nissl cells are the same as normal animals but at day 14th the cells have died and the number of viable cells has decrease significantly.

**Figure 4:**
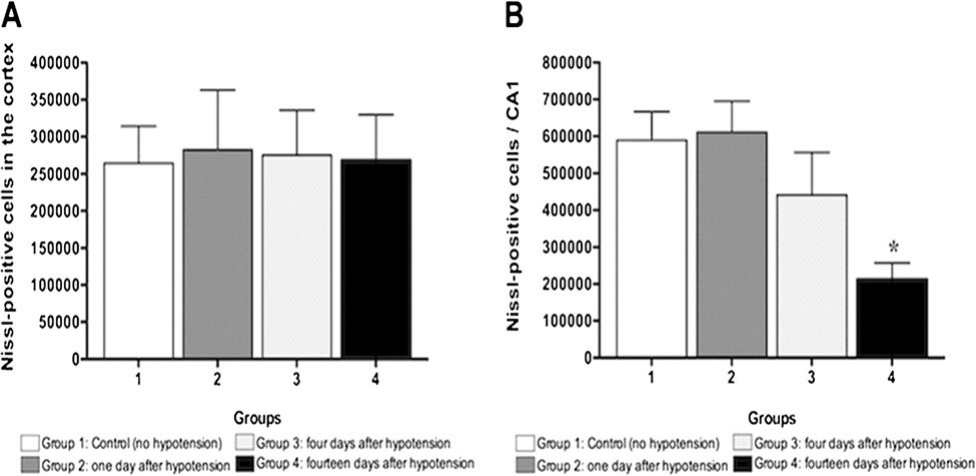
**Nissl-positive label in the (A) Cortex and (B) CA1 region, in control group, 1, 4 and 14 days following hypotension.** Nissl positive labeled was statistically significantly higher in the CA1 region of rats 14 day after hypotension as compared to control rats *p < 0.05. Nissl-positive label in the (**A**) Cortex and (**B**) CA1 region. Panel **A** shows the number of Nissl positive cells in frontal cortex. This is no difference in the number of cells at any of the time points. This suggests that the insult was not enough to cause cell dead. Panel **B** shows the number of Nissl positive cells in the CA1 hypocampal area. The number of Nissl positive cell decreased significantly 14 days after the insult. This finding suggest that the cells where damage with the insult and die progressively and this change becomes evident at day 14th.

On the other hand significant differences in cellular injury were seen between control rats and rats that received 3 min of hypotension on day one after the insult represented by numerous fluoro-jade C positive cells in the CA1 area of the hippocampus (p < 0.05). Figures 3–4. The Nissl stain, stains cells that have intact genetic materials by the time they where fixed, we can interpret that as "live cells". Fluoro jade C is a stain meant to stain broken genetic material at fixation time, this cells are either dead or degenerating. The tissue collection was done at 3 different time points. 1, 4 and 14 days. The cortex did not show any significant variations in the number of cells whit any of the dies. We interpreted this finding as a resistance to the cells to short periods of ischemia due to hypotension. The CA1 area of the hypocampus showed a gradual cellular lost represented as a decrease in the number of live cells (Nissl stain) at day 14 in comparison with day 1. This is correlated with the number of fluorojade positive cells that showed an important number of cells affected at day 1. In other words, the cells that were affected at day 1 (fluorojade C) are dead at day 14 for this reason the number of live cells decreases at day 14 (Nissl stain). This mechanism could explain why some patients, specially elderly patients show some cognitive impairment several days after surgery. The present observations suggest that exposure to repeated hypotensive episodes lead to hippocampal damage. Patients with hemodynamic TIAs, cerebral arteriosclerotic disease, or orthostatic hypotension may experience repeated nonfatal circulatory deficiencies (Yap et al. 2008). Our results suggest that in this rat hemorrhagic model, brief periods of hypotension result in neuronal damage or distress in the hippocampal CA1 region one day after insult. By day 14, surviving cells are significantly reduced in the hippocampus. We did not find any significant changes in cortical cells. Cellular lost is significant at day 14th and not at day 1 or 4th. We believe that apoptosis is an important factor that is responsible al least in part for this delay cellular lost.

In order to induce a hypotensive state, eight to twelve mL of blood were withdrawn to achieve a mean arterial blood pressure below 20 mmHg. The mean arterial blood pressure (MAP) for all the groups was 19.2 ± 1.1. Yamauchi (Yamauchi et al. 1990;1991) found histopathological changes after one week in the hippocampus of rats with an average MAP of 25 over a time frame of 2 or 3 min. Our data support the results shown by Yamauchi and colleagues and show that hystological damage continues to be present two week after the hypotensive insult.

The forty-eight point neurological scale did not show any statistically significant changes in motor skills. Interestingly, we found temporal paralysis in the upper body that lasted a few hours. At the time of the neurological evaluation, the paralysis had disappeared and could not be recorded as a positive finding. Although statistically insignificant, we found that after 24 hours of recovery several rats demonstrated palpebral ptosis. By day 4, all rats that had palpebral ptosis recovered. The dissection of the internal carotid artery typically manifests as an oculo-sympathetic palsy (myosis and palpebral ptosis) in humans (Eschmann et al. 2006). We placed the catheters in the common carotid artery but since the surgical area is small, it is possible that we had manipulated the internal carotid artery leading to this finding.

The passive avoidance paradigm has been used for memory evaluation. Bekker et al. (2009) used this paradigm in nitroglycerine-induced hypotensive and showed disruption in consolidation of long-term memory. In the present study we did not find any memory alterations. We hypothesize that this disparity is due to differences with the methods used to induce hypotension, as we used a hemorrhagic model to induce hypotension and Bekker and colleagues used nitroglycerine. Another difference between our studies is the time of hypotension. Bekker caused the hypotension early after the learning with the latest injection of nitroglycerine given 3 hours after the training. Our learning regimen took place 24 hours before hypotension and was tested twenty-four hours after hypotension. This study demonstrated that although there is damage in the hippocampus after 3 separate periods of profound hypotension, memories that are already consolidated were not affected.

## Conclusions

Our conclusions suggest that accidental cerebral blood circulation impairment, which may happen for example during surgical procedure with general procedure with general anesthesia in humans, needs to be considered carefully even in the absence of clearly evidenced functional signs of neurological damage.
